# Involvement of ADAMTS13 and von Willebrand factor in thromboembolic events in patients infected with SARS‐CoV‐2

**DOI:** 10.1111/ijlh.13244

**Published:** 2020-05-22

**Authors:** Albert Huisman, Robert Beun, Maaike Sikma, Jan Westerink, Nuray Kusadasi

**Affiliations:** ^1^ Department of Clinical Chemistry and Laboratory Medicine University Medical Center Utrecht and University Utrecht Utrecht The Netherlands; ^2^ Department of Intensive Care University Medical Center Utrecht and University Utrecht Utrecht The Netherlands; ^3^ Dutch Poisons Information Center University Medical Center Utrecht and University Utrecht Utrecht The Netherlands; ^4^ Department of Vascular Medicine University Medical Center Utrecht and University Utrecht Utrecht The Netherlands

Dear Editors,

Since early in 2020, the first people in Europe were infected with the SARS‐CoV‐2 virus leading to the COVID‐2019 pandemic. In March 2020, we admitted the first patients with a SARS‐CoV‐2 infection in our hospital. Many of these patients eventually end up at the intensive care unit (ICU). We and others have previously reported that patients infected with SARS‐CoV‐2 admitted to the ICU have a strikingly high rate of thromboembolic complications ranging between 25% and 35%.^1^, ^2^, ^3^ These patients have a hypercoagulable state at admission on the ICU and show mainly renal impairment as second organ involvement. Thrombotic microangiopathy (TMA) is one of the consequences of sepsis and may be caused by greatly reduced levels of “a disintegrin‐like metalloprotease with a thrombospondin type 1 motif, member 13” (ADAMTS13, von Willebrand cleaving protease). Severely reduced levels of ADAMTS13 (usually < 10%) cause accumulation of ultralarge von Willebrand factor (vWF) multimers, which can interact spontaneously with platelets, ultimately leading to microvascular thrombosis.^4^ The decrease of ADAMTS13 activity in consumptive coagulopathy has been shown to have a stronger relationship to platelet dynamics even before thrombocytopenia.^5^ There is also a probable risk of thrombosis induced by inflammation via vWF‐platelet axis.^6^, ^7^ To clarify whether ADAMTS13 and vWF are indeed involved in the pathophysiology of the thromboembolic complications seen in patients with SARS‐CoV‐2 infection, we measured ADAMTS13 and vWF levels in 12 patients with a clinical suspicion of microangiopathy (Table 1). A formal ethics review for this study was deemed unnecessary by the local Ethics Committee.

**TABLE 1:** Patient characteristics

Age (years) (mean, range)	61.8 (34‐80)
Gender (m/f)	10/2
Antithrombotic treatment	6 Patients: LMWH (dalteparin), prophylactic dose (1 dd 5000 IU) 1 Patient: LMWH (dalteparin), high prophylactic dose (2 dd 5000 IU) 1 Patient: LMWH: therapeutic dose 3 Patients: UFH 1 Patient: Arterial Thrombolysis
Ventilator use (n, %)	12 (100%)

Abbreviations: LMWH, low‐molecular‐weight heparin; UFH, unfractionated heparin.

Laboratory results for these patients are shown in Table 2. All laboratory assays were measured in the central ISO‐15189 accredited laboratory using an ACL Top 750 LAS coagulation analyzer (Werfen Diagnostics) using Coamatic factor VIII reagent (chromogenic factor VIII), QFA Thrombin reagent (fibrinogen), liquid antithrombin reagent (antithrombin), and D‐dimer HS500 reagent (D‐dimer) all obtained from Werfen diagnostics. Platelets were measured using an Alinity Hq hematology analyzer (Abbott Diagnostics). vWF antigen, vWF ristocetin cofactor activity (VWF:GPIbR), and ADAMTS13 activity were measured on an ACUstar chemiluminescence analyzer (Werfen); plasma creatinine concentration was measured on an Atellica chemistry analyzer (Siemens). The data in Table 2 show a low ADAMTS13 activity. In contrast, vWF levels and factor VIII levels are increased, probably due to activated endothelium.^8^

**TABLE 2:** Laboratory results

	Mean (SD)	Reference‐range
ADAMTS13 (IU/mL)	0.48 (0.14)	0.61‐1.31
ADAMTS13 < 0.10 IU/mL (n) (%)	0 (0%)	n.a.
vWF antigen (IU/mL)	4.08 (0.90)	0.60‐1.80
vWF:ag: ADAMTS13 ratio	8.5 (6.7)	0.5‐2.0
vWF ristocetin cofactor activity (IU/mL)	3.74 (1.05)	0.40‐1.80
Factor VIII (IU/mL)	4.30 (1.28)	0.60‐1.50
d‐dimer (mg/L)	19.5 (20.2)	<0.5
Fibrinogen (g/L)	6.7 (2.2)	2.0‐4.0
Antithrombin (IU/mL)	0.98 (0.21)	0.80‐1.20
Platelet count (10^9^/L)	398 (224)	150‐450
Creatinine (plasma, µmol/L)	180 (150)	49‐90
eGFR (mL/min/1.7 3m^2^)	51 (35)	>80
Patients with eGFR < 30 mL/min.1.73 m^2^ (%)	5 (42%)	n.a.

Note

Laboratory results for 12 patients infected with the SARS‐CoV‐2 virus with a clinical suspicion of microangiopathy admitted to the ICU.

Conversion factor for factor VIII, vWF, ADAMTS13, and antithrombin: 1.00 IU/mL = 100 IU/dL = 100%.

Abbreviations: eGFR, estimated glomerular filtration rate (according to the CKD‐EPI formula); n.a., not applicable; SD, standard deviation.

These data are in accordance with previously described results from patient with sepsis.^9^ The interaction between injured vessel wall, vWF, and ADAMTS 13 might be potentially relevant in the pathophysiological coagulation changes seen in sepsis patients with microthrombosis as seen in disseminated intravascular coagulation and microangiopathy.^4^, ^10^, ^11^ Low plasma levels of ADAMTS13 in sepsis patients are associated with worse outcome.^12^, ^13^ Because of the evidence indicating an association between vWF antigen: ADAMT13 ratio and risk on thrombosis, we also included this ratio to our measurements.^14^ As can be seen in Table 1, this ratio is high. Although the level of ADAMTS13 activity was not conclusive to confirm the microangiopathic state, the combination of low ADAMTS13 activity and high vWF levels might contribute to the microangiopathic state observed in these patients. We therefore suggest to include ADAMTS13 and vWF in the diagnostic workup of patient infected with SARS‐CoV‐2 when a microangiopathic state is suspected.

## CONFLICT OF INTEREST

The authors have no competing interests.

## AUTHOR CONTRIBUTION

All authors discussed the results and contributed to the final manuscript.
